# Supplementary material for: A global analysis of coral bleaching over the past two decades
Source: Nat Commun. 2019 Mar 20;10:1264. doi: 10.1038/s41467-019-09238-2 (PMC6427037; doi:10.1038/s41467-019-09238-2)
Supplement: Supplementary file 2 — Supplementary Information [file 41467_2019_9238_MOESM2_ESM.pdf]

Supplementary document for:

A global analysis of coral bleaching over the past two decades

Sully et al.

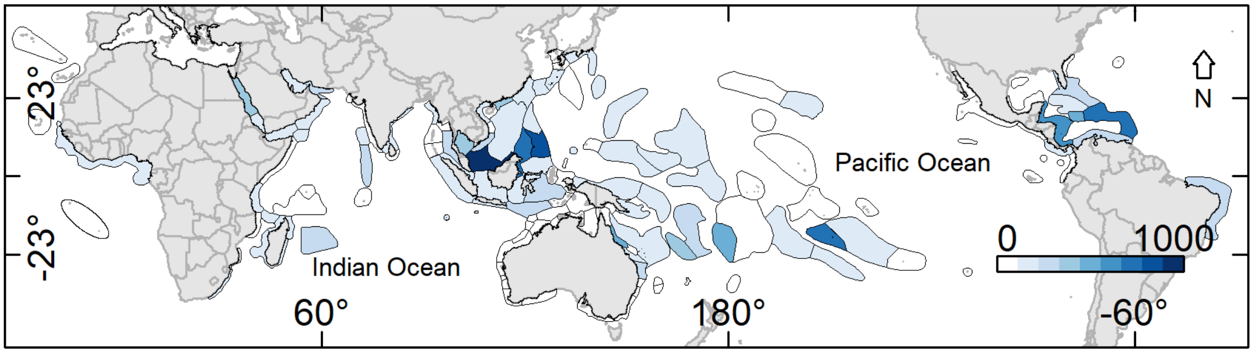

Supplementary Figure 1. Number of surveys in each ecoregion.

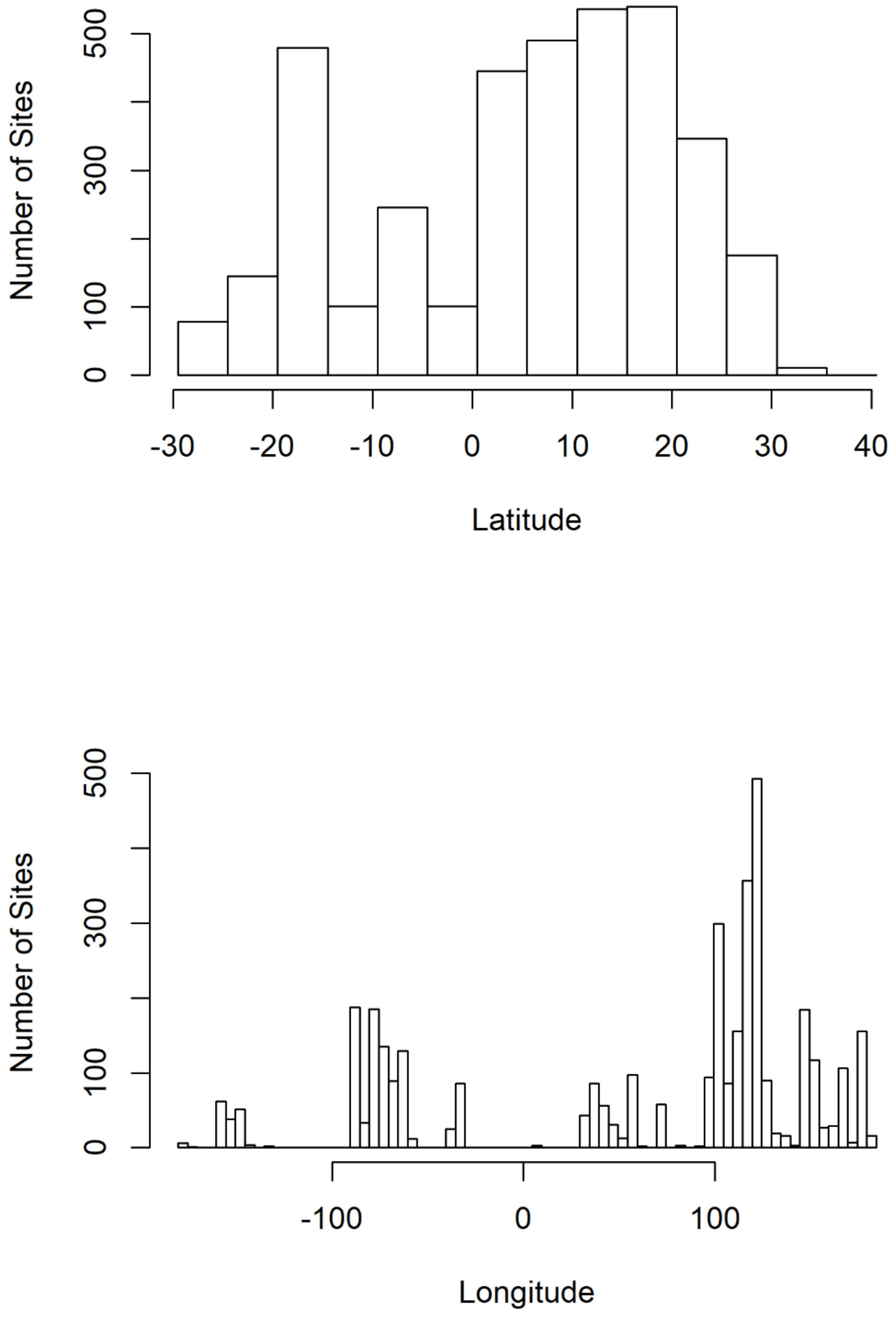

Supplementary Figure 2. Number of study sites by latitude and longitude.

Supplementary Table 1. Ecological parameter and temperature metric descriptions and sources. Ecological parameters and temperature metrics used to predict coral bleaching prevalence and intensity across reefs worldwide. CoRTAD variables are from NOAA's Coral Reef Temperature Anomaly Database (www.nodc.noaa.gov/sog/cortad/). All CoRTAD variables are provided on a grid cell basis, of approximately 4 km resolution. The study time frame for CoRTAD data is from 1982-2017, with time units of 1 week. Rate of SST change is calculated from NOAA's Optimum Interpolation (OI) SST, using years 1984 and 2017. Depth, latitude, and year were recorded by divers when each Reef Check study was conducted. Diversity data was provided by J.E.N. Veron.

Parameter	Metric Description	Metric Source
**Climatological SST (ClimSST)**	Climatological sea surface temperature (SST) based on weekly SSTs for the study time frame, created using a harmonics approach	www.nodc.noaa.gov/sog/cortad/Version6/
**Depth**	Provided in meters	Reef Check
**DHW (Degree Heating Weeks)**	Defined as 1 °C above the long-term average for the warmest month in a climatology	www.nodc.noaa.gov/sog/cortad/Version6/
**Diversity**	The number of coral species confirmed present in an ecoregion	J.E.N Veron (personal communication) and at www.coralsoftheworld.org/page/home/
**Rate_of_SST_change**	The average annual rate of sea surface temperature change in degrees Celsius	NOAA Optimum Interpolation (OI) Sea Surface Temperature (SST) V2. https://www.esrl.noaa.gov/psd/data/gridded/data.noaa.oisst.v2.html
**SST**	Mean sea surface temperature on a weekly basis	www.nodc.noaa.gov/sog/cortad/Version6/
**SST_Max**	Maximum sea surface temperature value over the entire time period	www.nodc.noaa.gov/sog/cortad/Version6/
**SST_Mean**	The mean SST value over the climatology	www.nodc.noaa.gov/sog/cortad/Version6/
**SST_stdev**	The standard deviation of the weekly SST over the entire time period	www.nodc.noaa.gov/sog/cortad/Version6/
**SSTA (Sea Surface Temperature Anomaly)**	Sea Surface Temperature Anomaly: weekly SST minus weekly climatological SST	www.nodc.noaa.gov/sog/cortad/Version6/
**SSTA_DHW**	Sea Surface Temperature Degree Heating Weeks: sum of previous 12 weeks when SSTA>=1 degree C	www.nodc.noaa.gov/sog/cortad/Version6/
**SSTA_DHW_Max**	The maximum SSTA_DHW over the entire time period	www.nodc.noaa.gov/sog/cortad/Version6/
**SSTA_DHW_Mean**	The mean SSTA_DHW over the entire time period	www.nodc.noaa.gov/sog/cortad/Version6/
**SSTA_DHW_stdev**	The standard deviation SSTA_DHW over the entire time period	www.nodc.noaa.gov/sog/cortad/Version6/
**SSTA_Frequency (SSTA_Freq)**	Sea Surface Temperature Anomaly Frequency: number of times over the	www.nodc.noaa.gov/sog/cortad/Version6/

	previous 52 weeks that SSTA >= 1 degree C	
**SSTA_Frequency_Max (SSTA_Freq_Max)**	The maximum SSTA_Frequency over the entire time period	www.nodc.noaa.gov/sog/cortad/Version6/
**SSTA_Frequency_stdev (SSTA_Freq_stdev)**	The standard deviation of SSTA_Frequency over the entire time period	www.nodc.noaa.gov/sog/cortad/Version6/
**SSTA_Max**	The maximum SSTA over the entire time period	www.nodc.noaa.gov/sog/cortad/Version6/
**SSTA_Mean**	The mean SSTA over the entire period	www.nodc.noaa.gov/sog/cortad/Version6/
**SSTA_stdev**	The Standard Deviation of weekly SST Anomalies over the entire time period	www.nodc.noaa.gov/sog/cortad/Version6/
**TSA (Thermal Stress Anomaly)**	Thermal Stress Anomaly: Weekly sea surface temperature minus the maximum of weekly climatological sea surface temperature	www.nodc.noaa.gov/sog/cortad/Version6/
**TSA_DHW**	Thermal Stress Anomaly (TSA) Degree Heating Week (DHW): Sum of previous 12 weeks when TSA >= 1 degree C	www.nodc.noaa.gov/sog/cortad/Version6/
**TSA_DHW_stdev**	The standard deviation of TSA_DHW over the entire time frame	www.nodc.noaa.gov/sog/cortad/Version6/
**TSA_DHW_Max**	The maximum TSA_DHW over the entire time frame	www.nodc.noaa.gov/sog/cortad/Version6/
**TSA_DHW_Mean**	The mean TSA_DHW over the entire time frame	www.nodc.noaa.gov/sog/cortad/Version6/
**TSA_Frequency (TSA_Freq)**	Thermal Stress Anomaly Frequency: number of times over previous 52 weeks that TSA >=1 degree C	www.nodc.noaa.gov/sog/cortad/Version6/
**TSA_Frequency_stdev (TSA_Freq_stdev)**	The standard deviation of frequency of thermal stress anomalies over the entire time frame	www.nodc.noaa.gov/sog/cortad/Version6/
**TSA_Frequency_Max (TSA_Freq_Max)**	The maximum TSA_Frequency over the entire time frame	www.nodc.noaa.gov/sog/cortad/Version6/
**TSA_Frequency_Mean (TSA_Freq_Mean)**	The mean TSA_Frequency over the entire time frame	www.nodc.noaa.gov/sog/cortad/Version6/
**TSA_Max**	The maximum TSA over the entire time frame	www.nodc.noaa.gov/sog/cortad/Version6/
**TSA_Mean**	The mean TSA over the entire time frame	www.nodc.noaa.gov/sog/cortad/Version6/
**TSA_stdev**	The standard deviation of TSA over the entire time frame	www.nodc.noaa.gov/sog/cortad/Version6/

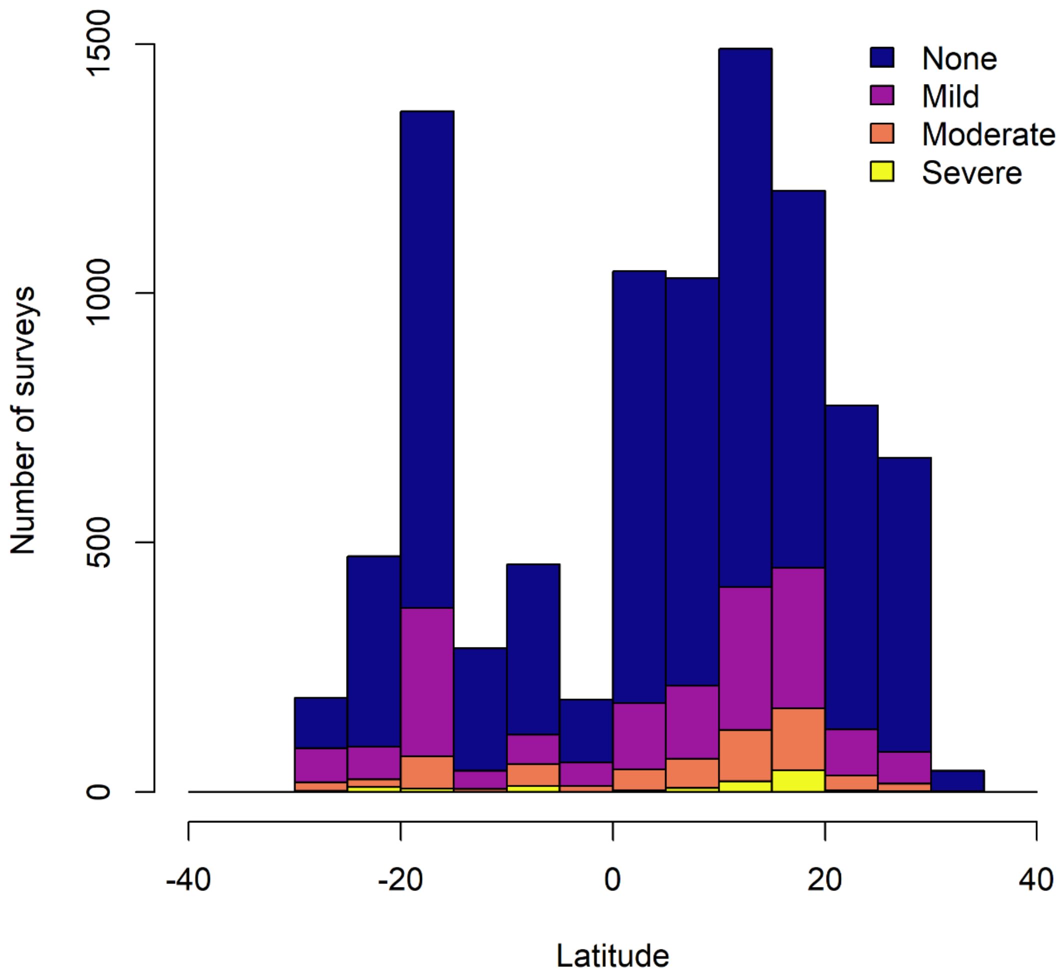

Supplementary Figure 3. Bleaching prevalence by latitude. Bleaching prevalence by latitude, where the blue indicates no bleaching, purple is mild bleaching (1-10% bleached), peach is moderate bleaching (10-50% bleached), and yellow is severe bleaching (>50% bleached).

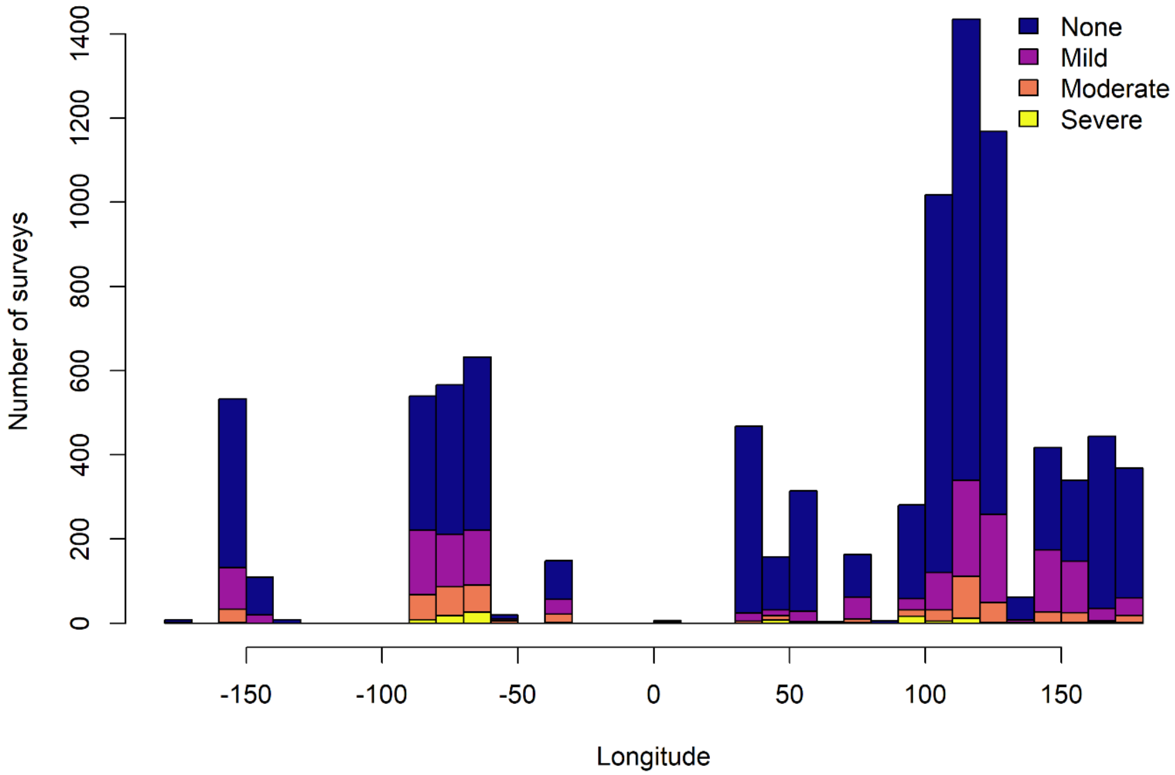

Supplementary Figure 4. Bleaching prevalence by longitude. Bleaching prevalence by longitude, where the blue indicates no bleaching, purple is mild bleaching (1-10% bleached), pink is moderate bleaching (10-50% bleached), and yellow is severe bleaching (>50% bleached).

Supplementary Table 2. Kolmogorov-Smirnov test results comparing bleaching probabilities at latitudes 15 through 20 degrees North and South with bleaching probabilities at other latitudes.

Latitudes in comparison	Bleaching levels in comparison	D	p-value
15˚–20˚ N & S, compared with all others	bleaching vs nonbleaching	0.10736	<0.001
15˚–20˚ N & S, compared with all others	mild bleaching vs moderate or severe bleaching	0.0059019	1
15˚–20˚ N & S, compared with all others	mild or moderate bleaching vs severe bleaching	0.019203	0.9913
15˚–20˚ N & S, compared with 10˚–15˚ N & S	bleaching vs nonbleaching	0.096466	<0.001

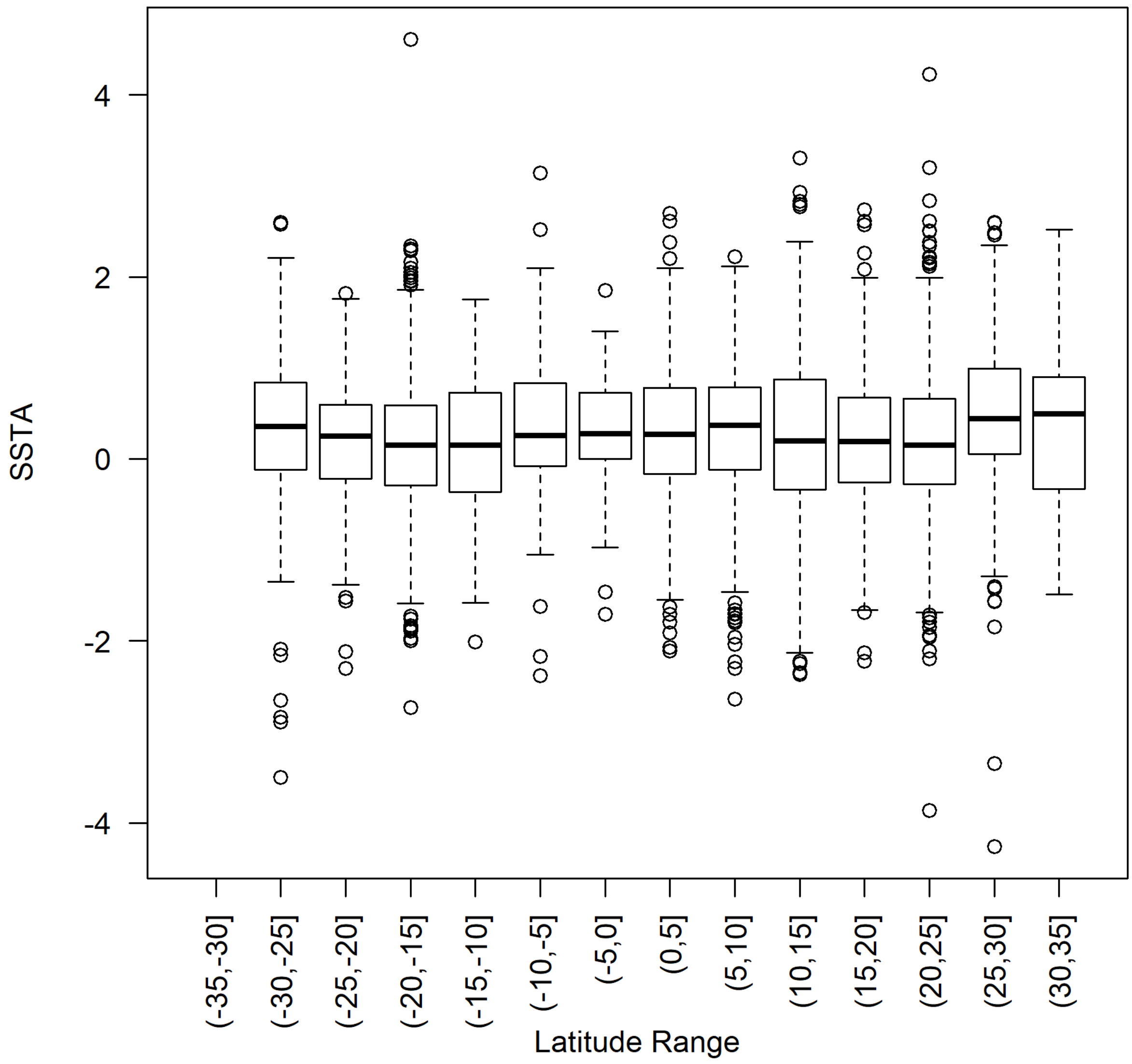

Supplementary Figure 5. Sea surface temperature anomalies by latitude. Sea-surface temperature anomalies (SSTA), which is the weekly sea-surface temperature minus the weekly climatological sea-surface temperature, by latitude. The thick center line is the median value, the bounds of the box are the interquartile range (25% and 75%), the whiskers are the 95% range, and open circles are surveys falling outside the 95% range.

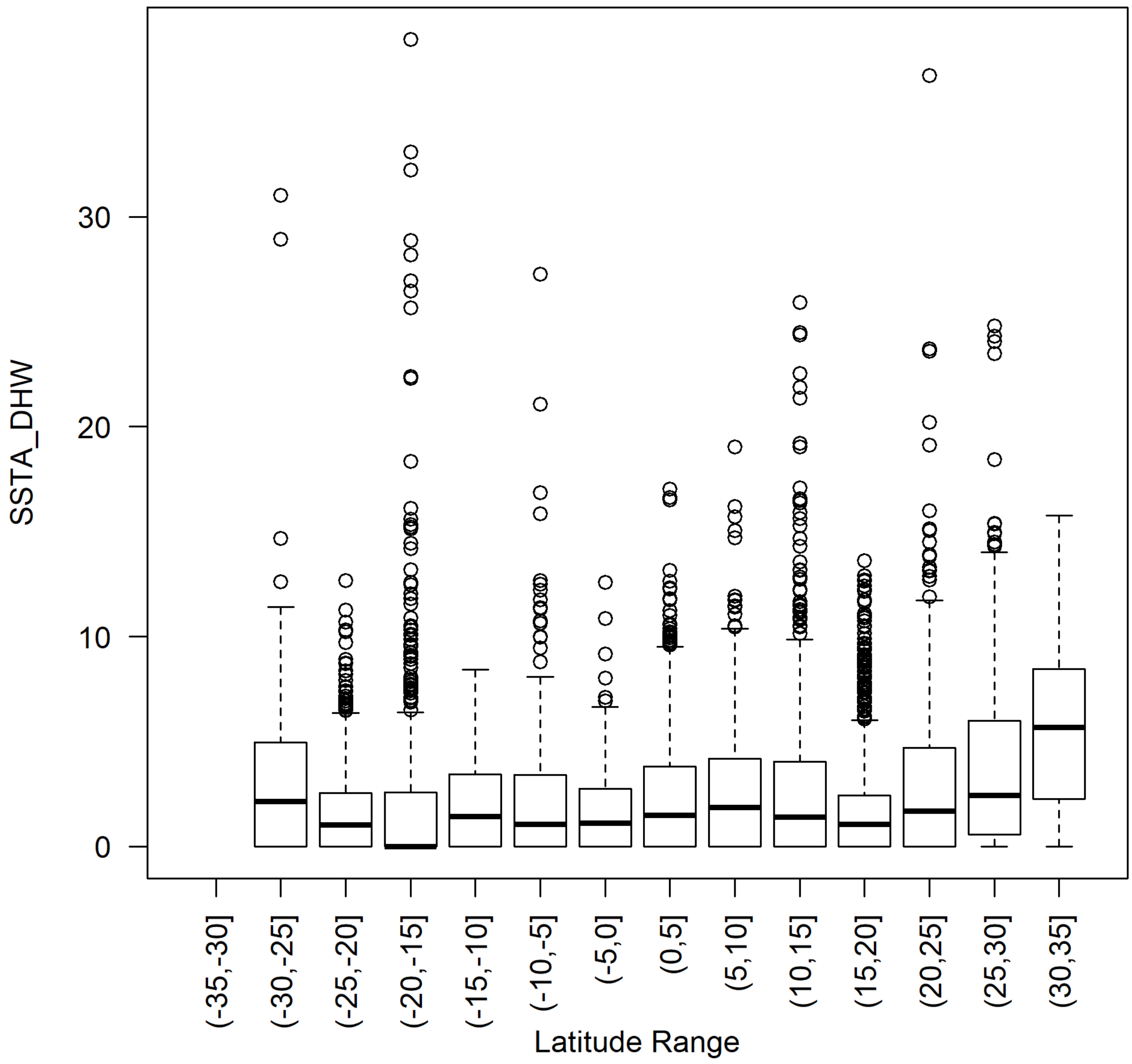

Supplementary Figure 6. Degree heating weeks by latitude. The sum of the previous 12 week, or Degree Heating Weeks (DHW) when SSTA >= 1 degree C, where SSTA is the weekly sea-surface temperature minus weekly climatological sea-surface temperature, by latitude. The thick center line is the median value, the bounds of the box are the interquartile range (25% and 75%), the whiskers are the 95% range, and open circles are surveys falling outside the 95% range.

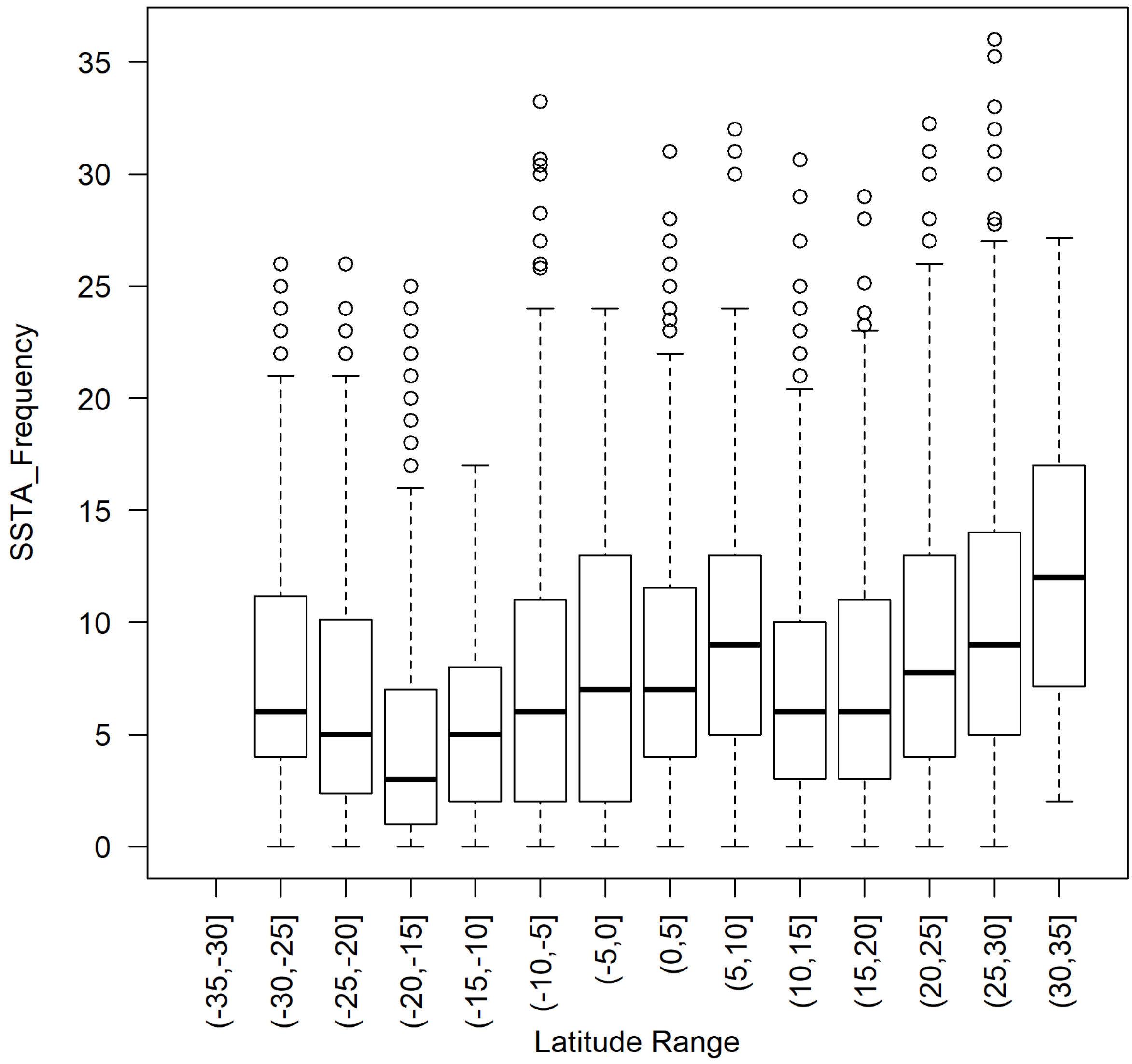

Supplementary Figure 7. Frequency of sea surface temperature anomalies by latitude. The frequencies of sea-surface temperature anomalies (SSTA), where SSTA is the weekly sea-surface temperature minus weekly climatological sea-surface temperature, by latitude. The thick center line is the median value, the bounds of the box are the interquartile range (25% and 75%), the whiskers are the 95% range, and open circles are surveys falling outside the 95% range.

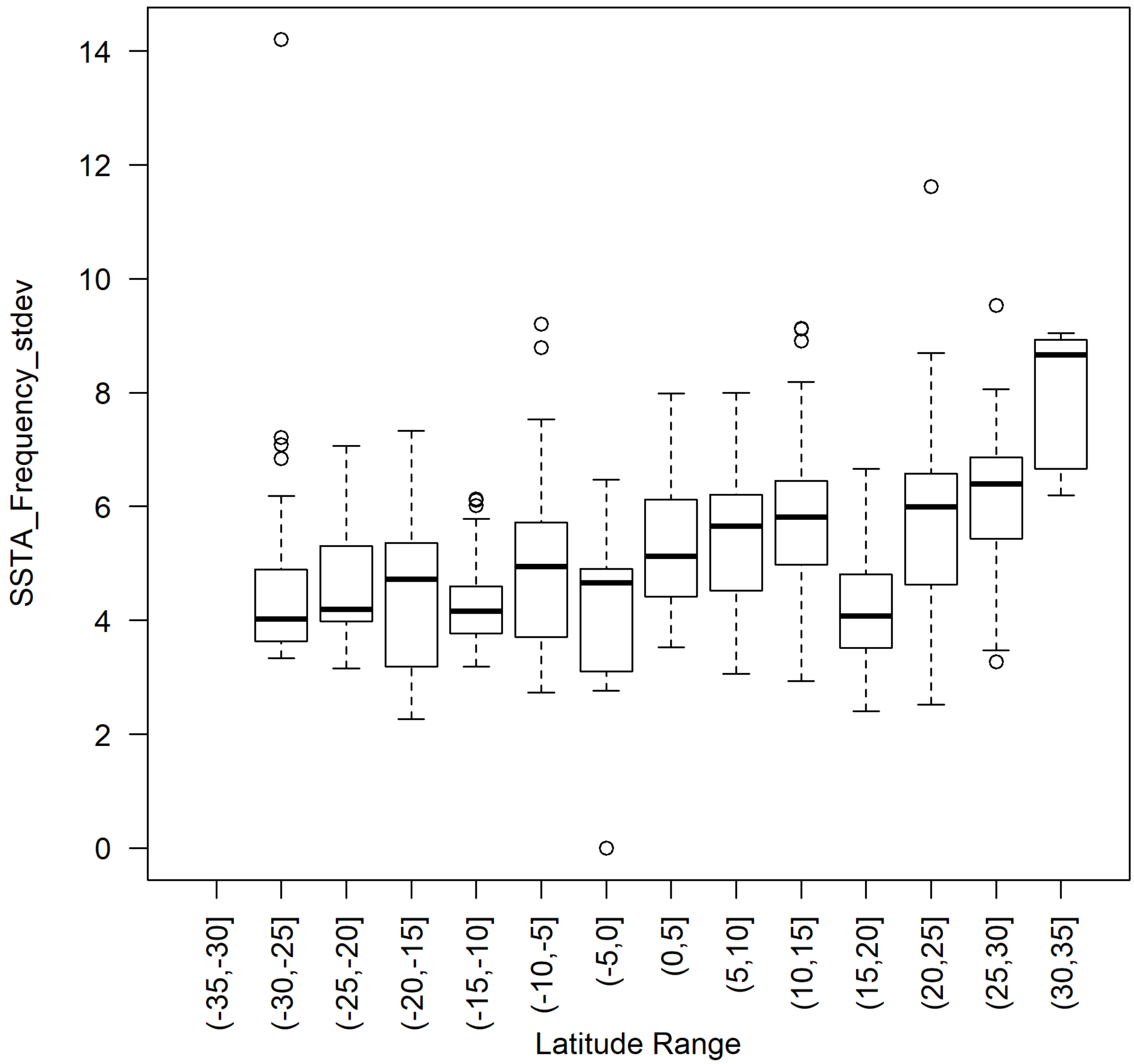

Supplementary Figure 8. Standard deviation of the frequency of sea surface temperature anomalies by latitude. The standard deviations of the frequencies of sea-surface temperature anomalies (SSTA), where SSTA is the weekly sea-surface temperature minus weekly climatological sea-surface temperature, by latitude. The thick center line is the median value, the bounds of the box are the interquartile range (25% and 75%), the whiskers are the 95% range, and open circles are surveys falling outside the 95% range.

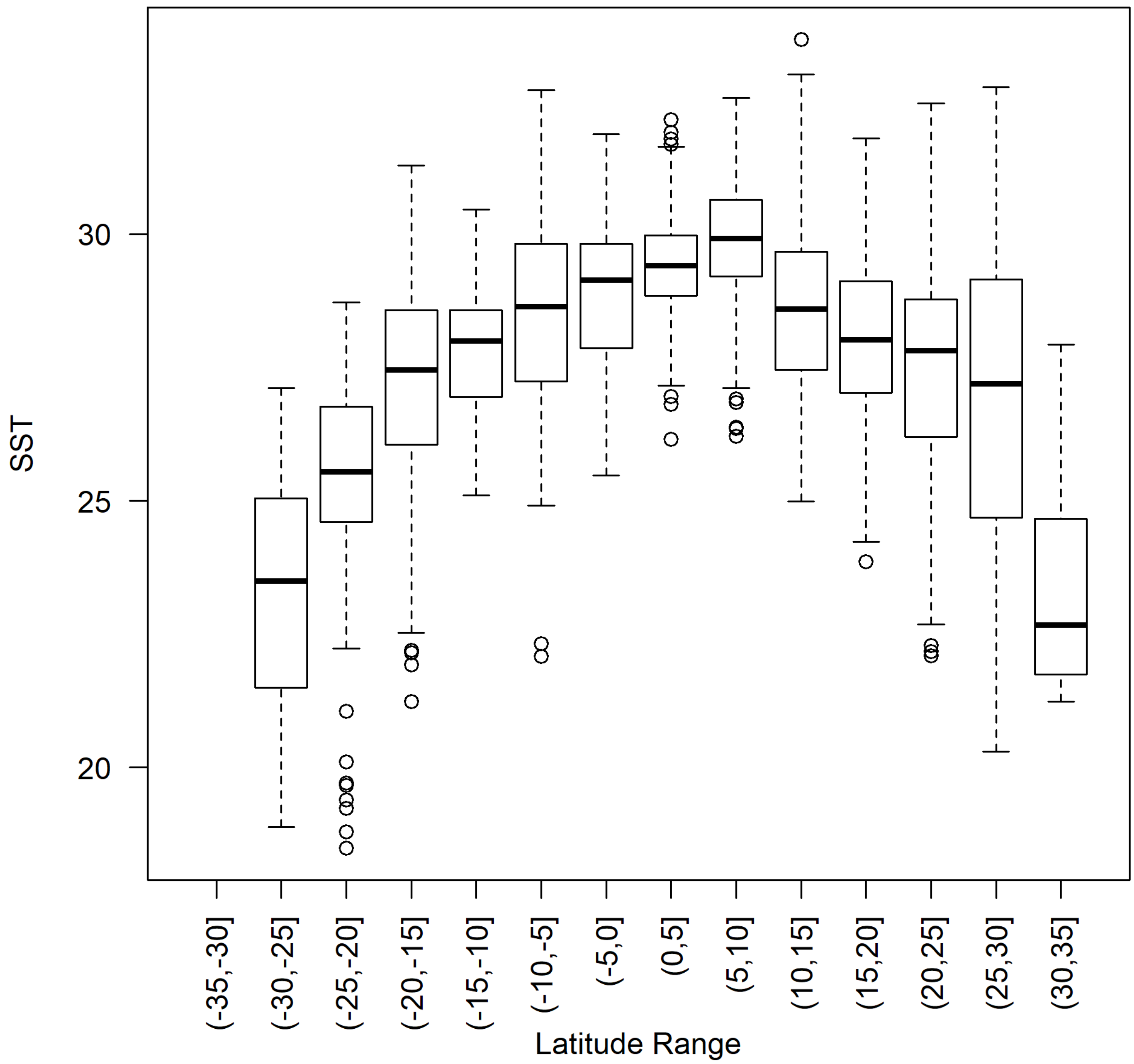

Supplementary Figure 9. Sea surface temperature by latitude. Sea-surface temperatures in degrees Celsius by latitude. The thick center line is the median value, the bounds of the box are the interquartile range (25% and 75%), the whiskers are the 95% range, and open circles are surveys falling outside the 95% range.

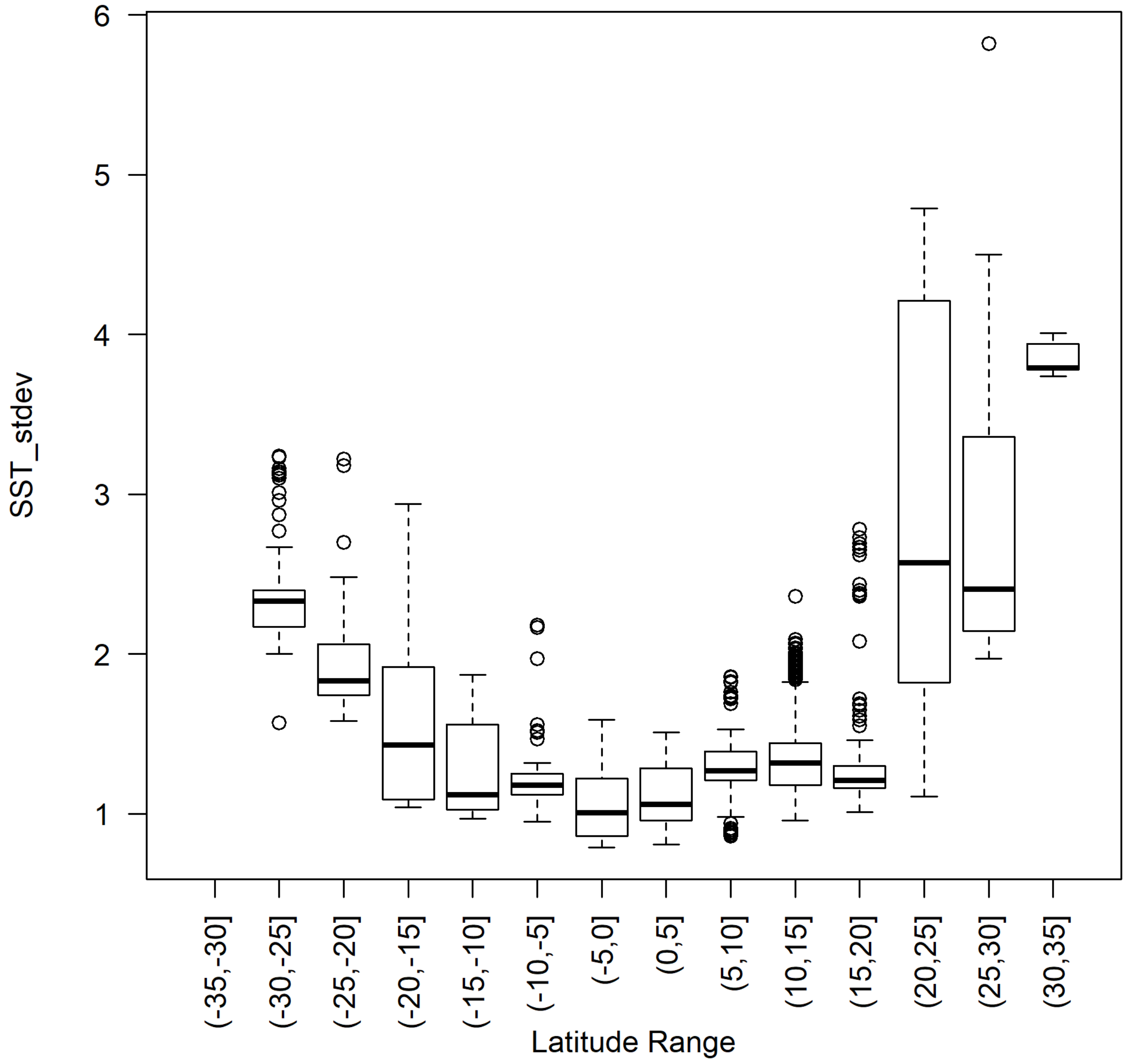

Supplementary Figure 10. Standard deviation of sea surface temperature by latitude. The standard deviations of sea-surface temperatures by latitude. The thick center line is the median value, the bounds of the box are the interquartile range (25% and 75%), the whiskers are the 95% range, and open circles are surveys falling outside the 95% range.

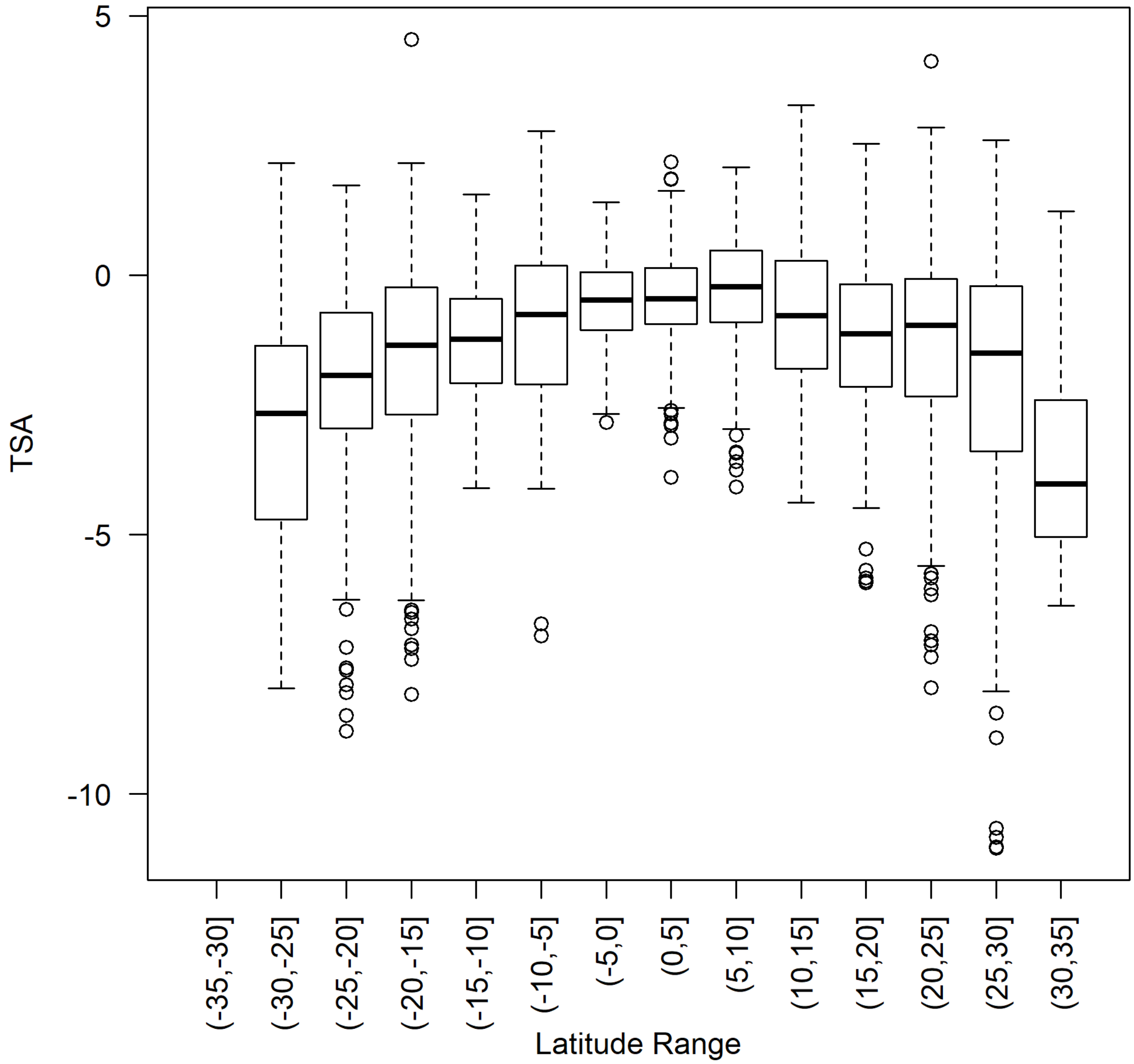

Supplementary Figure 11. Thermal stress anomalies by latitude. Thermal stress anomaly (TSA), which is the weekly sea-surface temperature minus the maximum weekly climatology, by latitude. The thick center line is the median value, the bounds of the box are the interquartile range (25% and 75%), the whiskers are the 95% range, and open circles are surveys falling outside the 95% range.

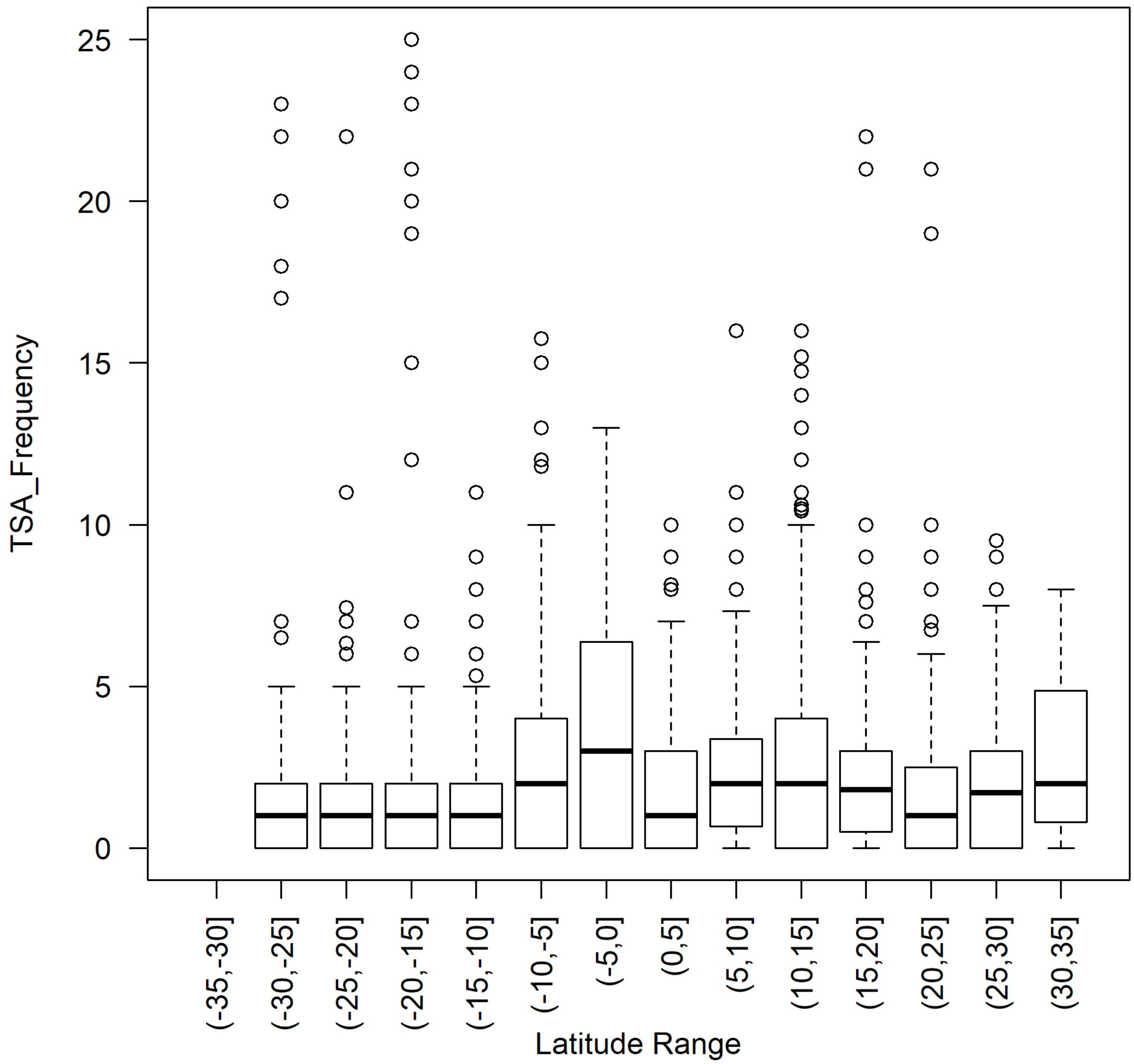

Supplementary Figure 12. Frequency of thermal stress anomalies by latitude. The frequency of thermal-stress anomalies, which is the number of times over previous 52 weeks that the thermal stress anomaly (TSA) >=1 degree C, and where TSA is the weekly sea surface temperature minus the maximum weekly climatological sea surface temperatures, by latitude. The thick center line is the median value, the bounds of the box are the interquartile range (25% and 75%), the whiskers are the 95% range, and open circles are surveys falling outside the 95% range.

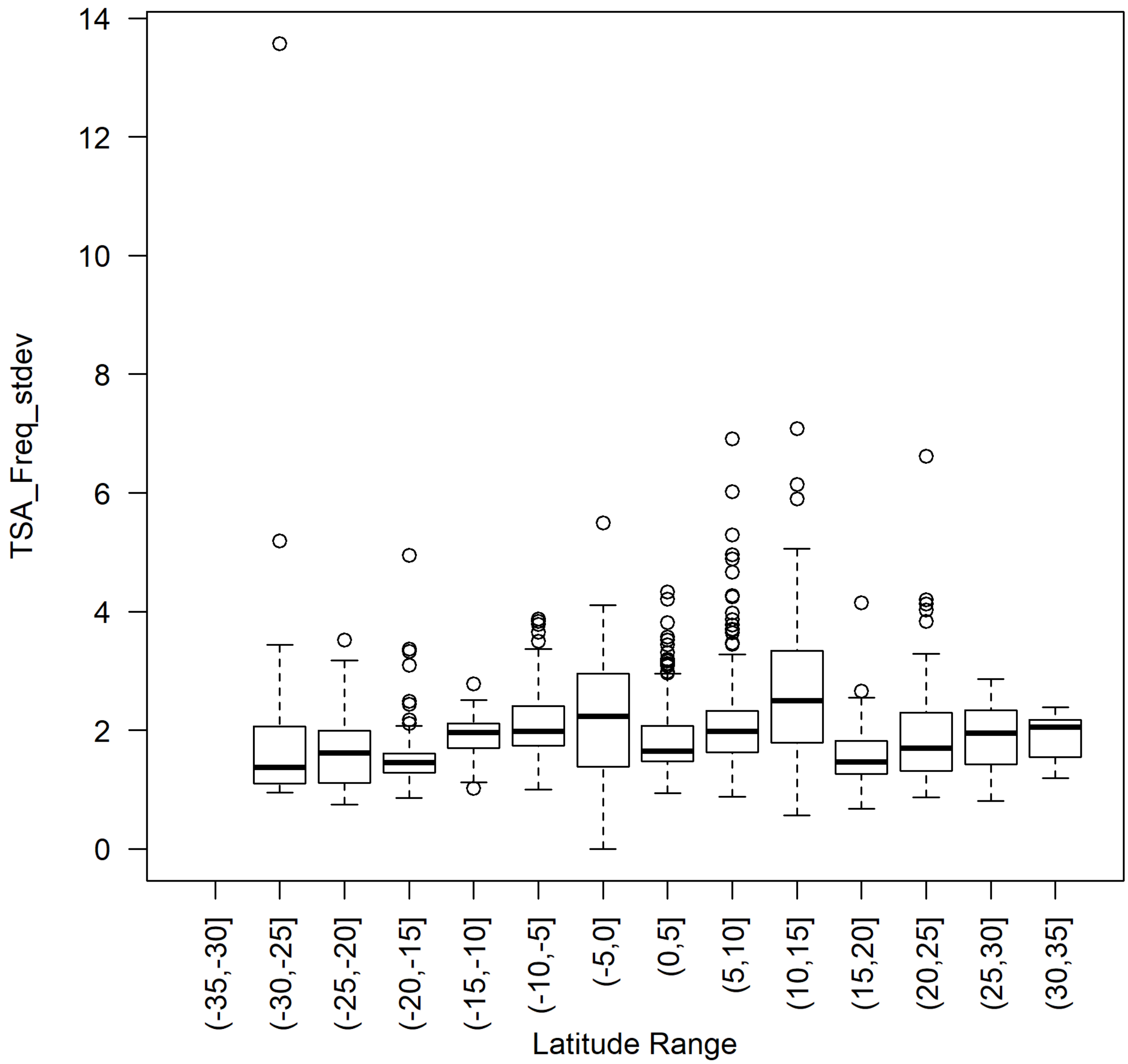

Supplementary Figure 13. Standard deviation of the frequency of thermal stress anomalies by latitude. The standard deviations of thermal-stress anomaly (TSA) Frequency (TSA_Frequency is the number of times over previous 52 weeks that TSA >=1 degree C), over the entire time period (1998 to 2017), by latitude. The thick center line is the median value, the bounds of the box are the interquartile range (25% and 75%), the whiskers are the 95% range, and open circles are surveys falling outside the 95% range.

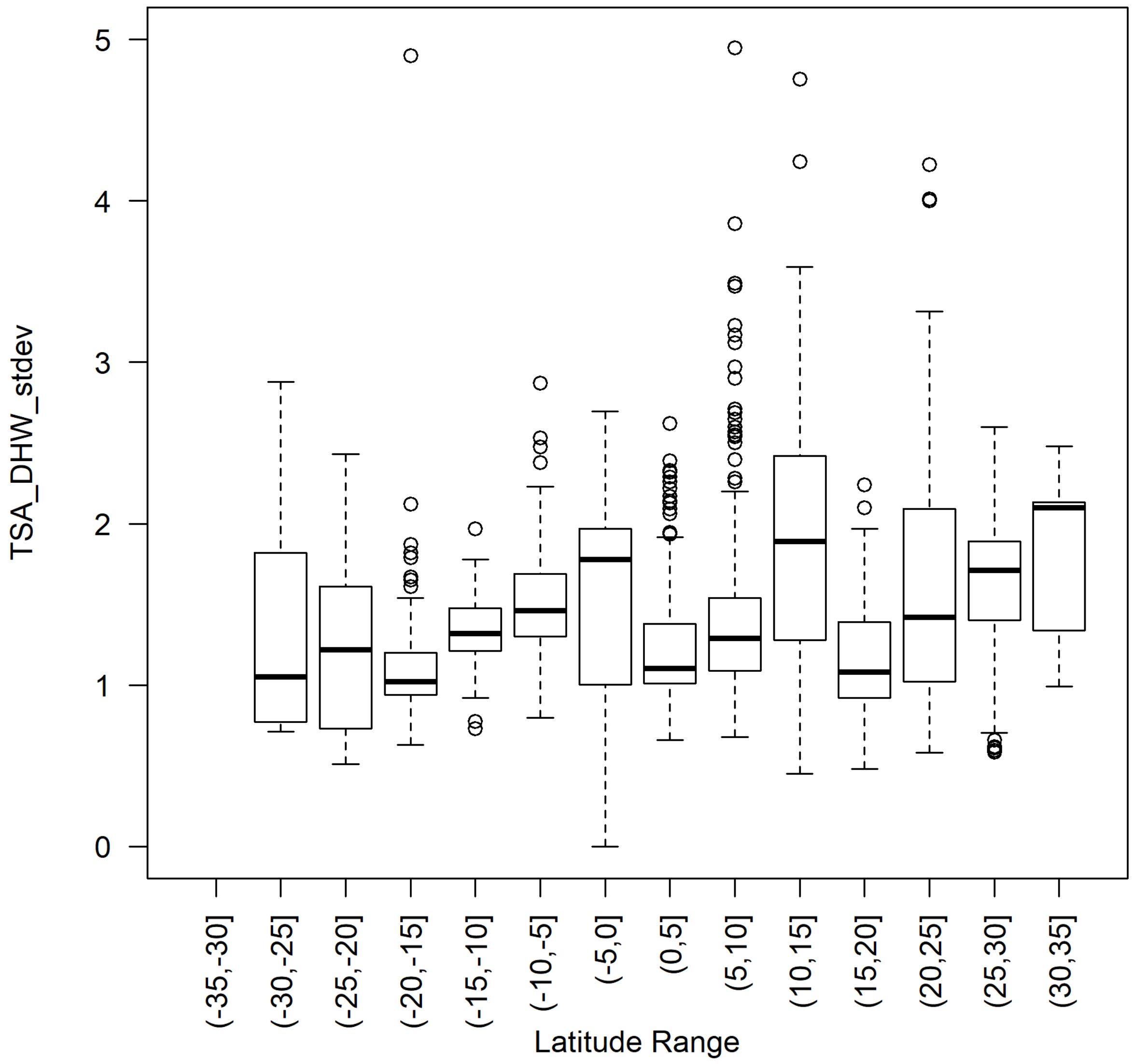

Supplementary Figure 14. Standard deviation of thermal stress anomaly degree heating weeks by latitude. Thermal Stress Anomaly (TSA) Degree Heating Weeks (DHW) Standard Deviation, by latitude. The thick center line is the median value, the bounds of the box are the interquartile range (25% and 75%), the whiskers are the 95% range, and open circles are surveys falling outside the 95% range.

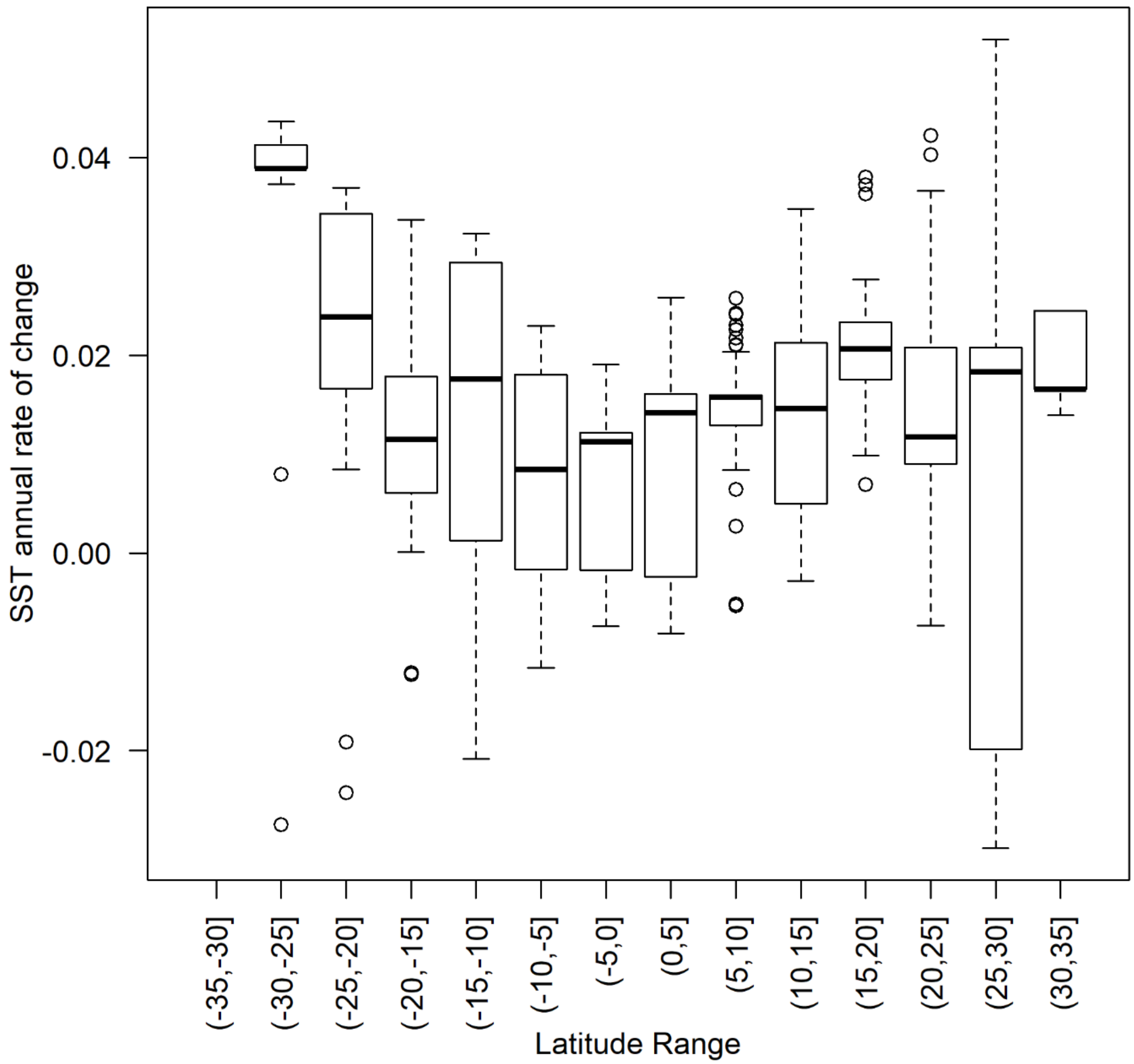

Supplementary Figure 15. Sea surface temperature annual rate of change by latitude. Sea Surface Temperature (SST) annual rate of change, which is calculated from the mean SST of 1984 and the mean SST of 2017, by latitude. The thick center line is the median value, the bounds of the box are the interquartile range (25% and 75%), the whiskers are the 95% range, and open circles are surveys falling outside the 95% range.

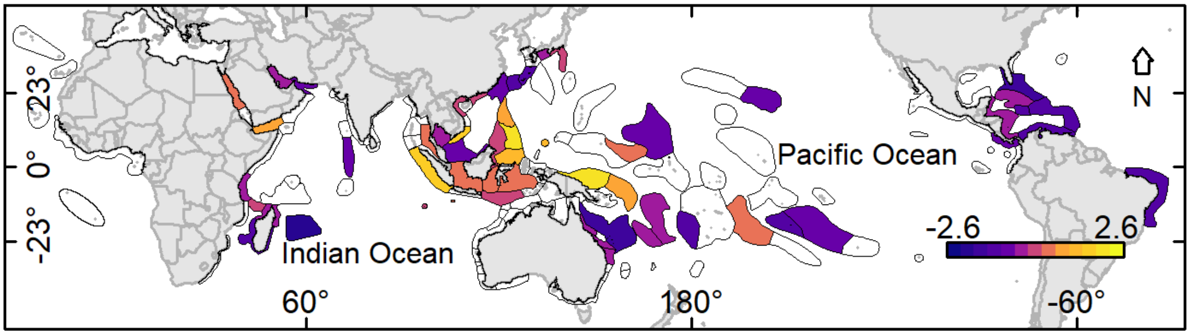

Supplementary Figure 16. Standard deviation of frequency of thermal stress anomalies. Standard deviation of frequency of thermal stress anomalies (TSA_Freq_stdev), for data from 1998 to 2017. The value of each ecoregion is displayed as the number of standard deviations it differs from the mean of all ecoregions. Positive values are displayed in red and negatives values are displayed in blue.

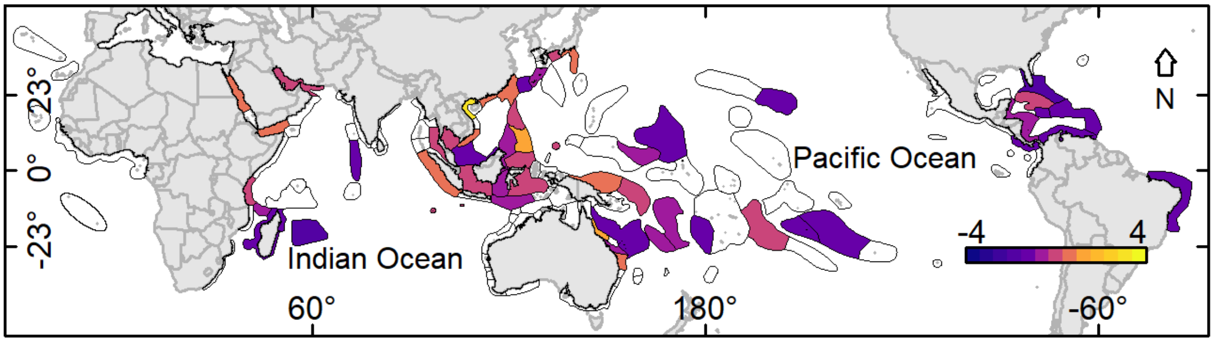

Supplementary Figure 17. Standard deviation of thermal stress anomaly degree heating weeks. Standard deviation of thermal stress anomaly degree heating weeks (TSA_DHW_stdev), for data from 1998 to 2017. The value of each ecoregion is displayed as the number of standard deviations it differs from the mean of all ecoregions. Positive values are displayed in red and negatives values are displayed in blue.

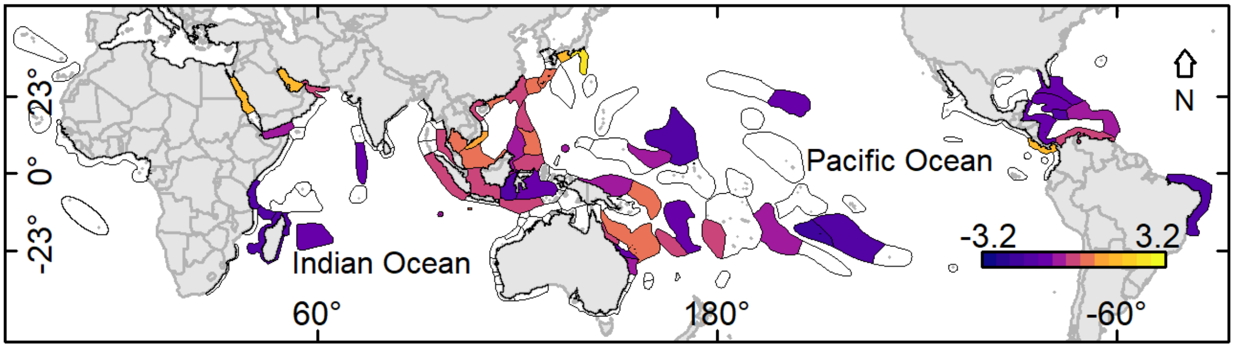

Supplementary Figure 18. Standard deviation of frequency of sea surface temperature anomalies. Standard deviation of frequency of sea surface temperature anomalies (SSTA_Freq_stdev), for data from 1998 to 2017. The value of each ecoregion is displayed as the number of standard deviations it differs from the mean of all ecoregions. Positive values are displayed in red and negatives values are displayed in blue.

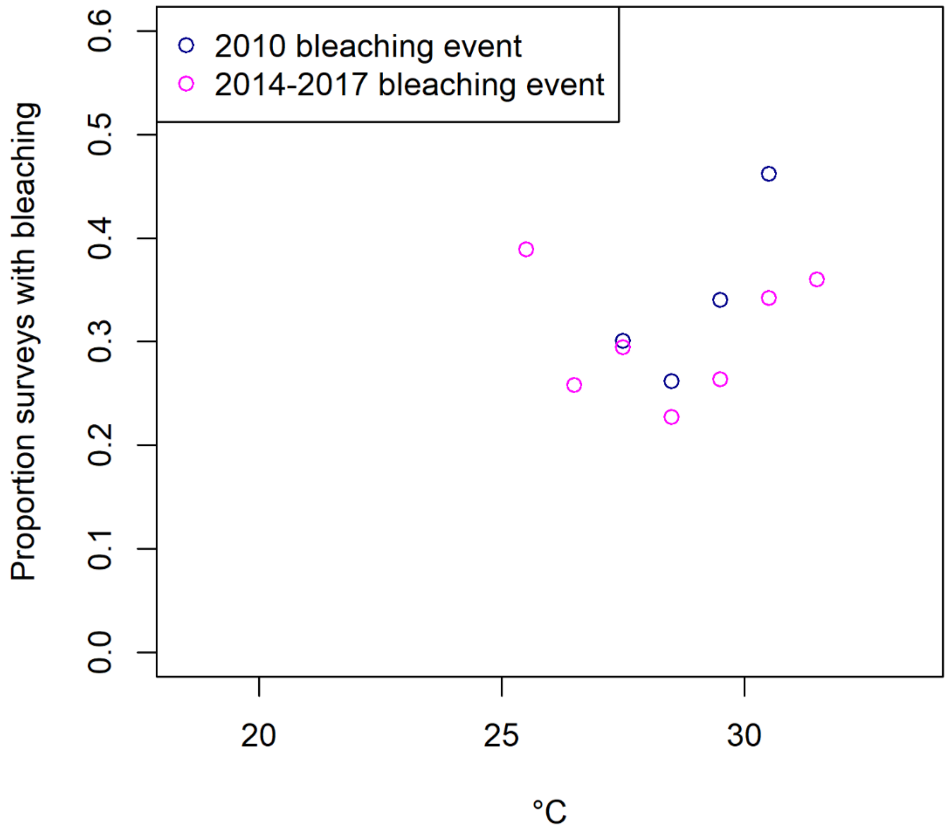

Supplementary Figure 19. Proportion of surveys with bleaching at temperature. The proportion of surveys at a given temperature that found coral bleaching during the 2010 global bleaching event compared to the 2014-2017 global bleaching event. Bin size is 1 degree Celsius, and each bin was required to have 60 surveys in order to be included. Blue dots indicate the 2010 bleaching event, and magenta dots indicate the 2014-2017 bleaching event.

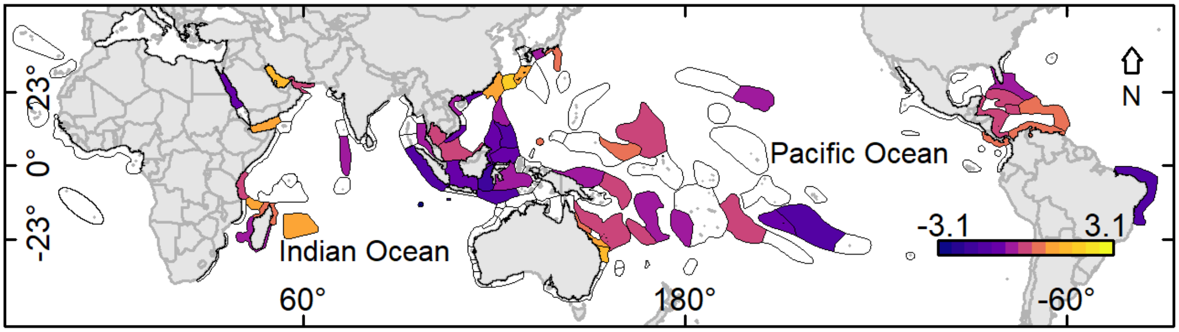

Supplementary Figure 20. Annual rate of sea surface temperature change. Annual rate of sea surface temperature change, for data from 1998 to 2017. The value of each ecoregion is displayed as the number of standard deviations it differs from the mean of all ecoregions. Positive values are displayed in red and negatives values are displayed in blue.

Supplementary Table 3. Number of surveys in each ecoregion. Ecoregion name and the number of Reef Check surveys. 8797 Reef Check studies were located within ecoregion boundaries. All other Reef Check surveys (418 surveys at 153 sites) that were not located within the ecoregion boundaries were not included in this table.

**Ecoregion Name**	**Number of Studies**
North and central Red Sea	230
South Red Sea	9
Gulf of Aden	26
North-west Arabian Sea	2
Gulf of Oman	120
Persian Gulf	17
Socotra Archipelago	7
East Somali coast	0
Kenya and Tanzania coast	30
North Mozambique coast	1
South Mozambique coast	0
Eastern coast South Africa	3
Mayotte and Comoros	73
Northern Seychelles	0
Southern Seychelles	0
North Madagascar	11
South Madagascar	25
Mascarene Islands	135
Chagos Archipelago	0
Maldive Islands	161
Lakshadweep Islands	2
Gulf of Kutch, India	0
South and west India coast	0
South Sri Lanka	0
North Sri Lanka and east India	3
North Myanmar and Bangladesh	2
Gulf of Martaban, Myanmar	0
Andaman Sea	150
Andaman Islands	0
Nicobar Islands	0
West Sumatra	26
Strait of Malacca	6
Java Sea	52
South Java	7
Lesser Sunda Islands and Savu Sea	121
Makassar Strait, Indonesia	40

Gulf of Tomini, Indonesia	5
Banda Sea and Molucca Islands	161
North Arafura Sea Islands	0
Coastal south-west Papua	0
Cenderawasih Bay, Papua	6
Birds Head Peninsula, Papua	1
Halmahera, Indonesia	0
Celebes Sea	67
Sulu Sea	579
South-east Philippines	630
North Philippines	76
South China Sea	5
Sunda Shelf, south-east Asia	929
Gulf of Thailand	221
South Vietnam	132
Central Vietnam	0
North Vietnam	34
Hainan, South China Sea	0
Hong Kong	278
Taiwan and coastal China	98
South Ryukyu Islands, Japan	72
North Ryukyu Islands, Japan	28
South-east Kyushu, Japan	0
North Kyushu and South Korea	0
Shikoku, Japan	28
Honshu, Japan	14
Ogasawara Islands, Japan	4
Okinotorishima, Japan	0
Marianas	8
Palau	12
Helen Reef	0
Yap Islands, Micronesia	0
Caroline Islands, Micronesia	2
Pohnpei and Kosrae, Micronesia	70
Marshall Islands	12
Gilbert Islands, west Kiribati	2
Bismarck Sea, New Guinea	45
Solomon Islands and Bougainville	57
Milne Bay, Papua New Guinea	2
Gulf of Papua, Papua New Guinea	0
Torres Strait and far northern Great Barrier Reef	0

Central and northern Great Barrier Reef	378
Coral Sea	11
Pompey and Swain Reefs, south-east Great Barrier Reef	0
Southern Great Barrier Reef	58
Moreton Bay, eastern Australia	183
Solitary Islands, eastern Australia	0
Central New South Wales, south-eastern Australia	0
South-east Australia	0
Lord Howe Island, east Australia	0
Elizabeth and Middleton Reefs, eastern Australia	0
Norfolk Island	0
Recherche Archipelago, south-west Australia	0
Geographe Bay, south-west Australian coast	0
Direction Bank, south-west Australian coast	0
Houtman Abrolhos Islands, west Australia	1
Shark Bay, west Australia	0
Ningaloo Reef and coastal north-west Australia	1
Rowley Shoals, west Australia	0
Scott Reef, west Australia	0
Kimberley Coast, north-west Australia	0
Ashmore Reef, north-west Australia	0
Darwin, north Australia	0
Arnhem Land, north Australia	0
Arafura Sea	0
Timor Sea	0
Joseph Bonaparte Gulf, north-west Australia	0
Gulf of Carpentaria, northern Australia	0
Christmas Island, Indian Ocean	20
Cocos Keeling Atolls, Indian Ocean	32
New Caledonia	201
Vanuatu	161
Kermadec Islands, south Pacific	0
Phoenix Islands, central Kiribati	0
Fiji	373
Samoa, Tuvalu and Tonga	1
Cook Islands, south-west Pacific	20
Austral Islands, French Polynesia	5
Society Islands, French Polynesia	516
Western Tuamotu Archipelago, central Pacific	44
Pitcairn and south-east Tuamoto Archipelago	0
Line Islands, south-east Kiribati	0

Marquesas Islands, French Polynesia	0
Line Islands, north-east Kiribati	0
Johnston Atoll, north central Pacific	0
North-west Hawaii	0
Eastern Hawaii	64
Easter Island, south central Pacific	0
Gulf of California	0
Western Mexico and Revillagigedo Islands	0
Clipperton Atoll, eastern Pacific	0
Guatemala, El Salvador and Nicaragua, Pacific coast	0
Costa Rica and Panama, Pacific coast	18
Colombia, Ecuador and Chile, Pacific coast	0
Isla de Malpelo, Colombia	0
Cocos Island, Costa Rica	0
Galapagos Islands	0
Flower Garden Banks, Gulf of Mexico	0
Bay of Campeche, Yucatan, Gulf of Mexico	0
Belize and west Caribbean	420
Netherlands Antilles and south Caribbean	173
Hispaniola, Puerto Rico and Lesser Antilles	581
Jamaica	344
Cuba and Cayman Islands	25
Bahamas and Florida Keys	176
North Florida to North Carolina	0
Bermuda	0
Brazil	148
Central Atlantic	0
Gulf of Guinea to Sierra Leone	6
Cape Verde Islands	0
Canary Islands	0
Madeira and Azores Islands	0
Mediterranean	0

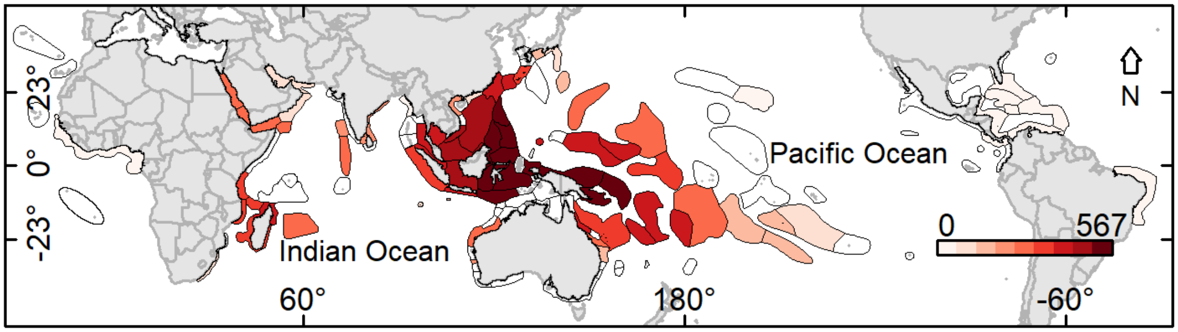

Supplementary Figure 21. Coral diversity. Diversity is displayed as the number of coral species confirmed present in each ecoregion ranging from 0 (white) to 567 (dark red).

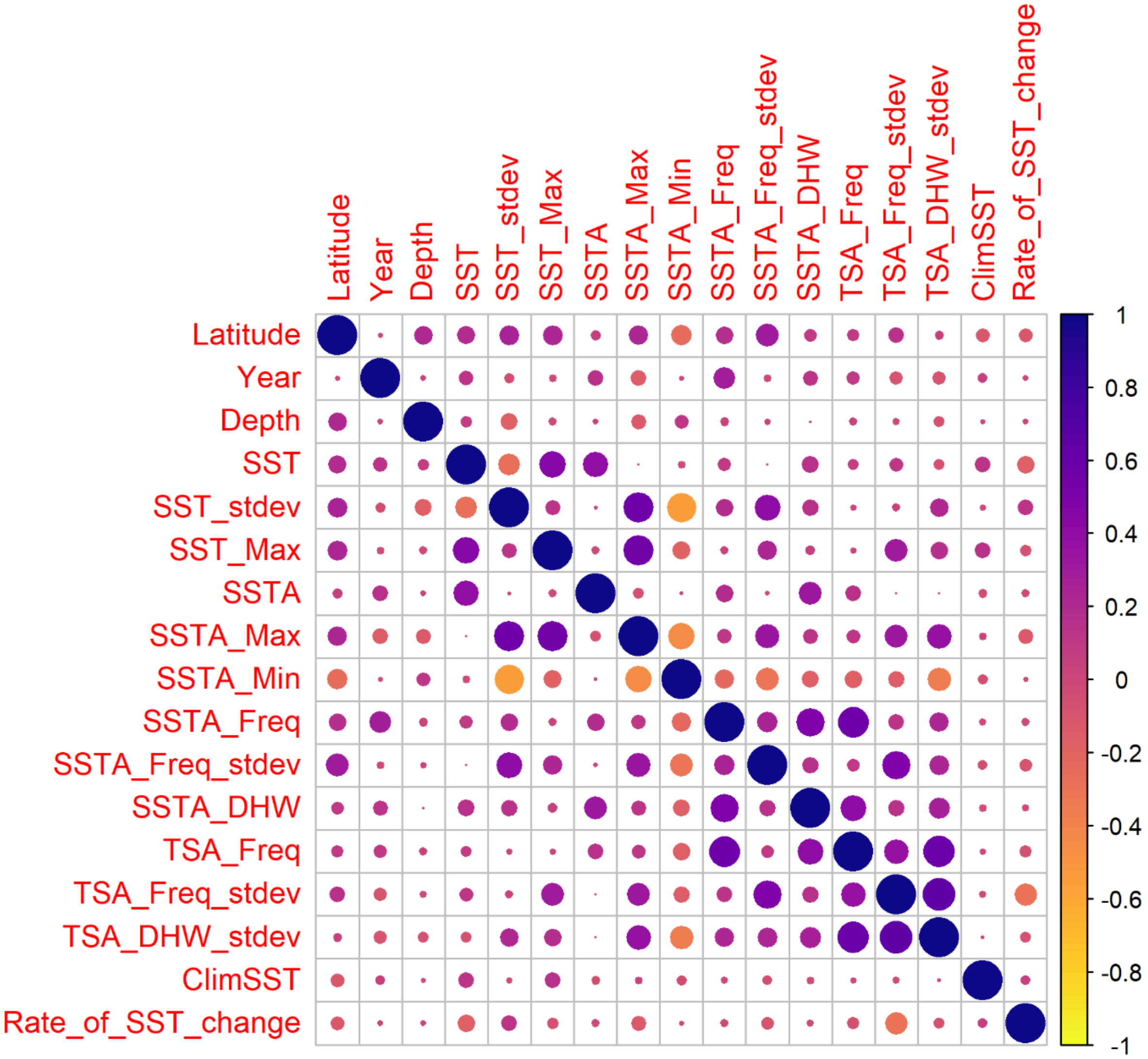

Supplementary Figure 22. Covariate correlations. Correlation plot of variables. Blue indicates that variables are positively correlated, magenta indicates no correlation, and yellow indicates that variables are negatively correlated. Shade of the color and size of the circles indicate the strength of the correlation between variables.
